# Ding Chuan Tang Attenuates Airway Inflammation and Eosinophil Infiltration in Ovalbumin-Sensitized Asthmatic Mice

**DOI:** 10.1155/2021/6692772

**Published:** 2021-09-20

**Authors:** Jason Ma, Ming-Xun Liu, Li-Chen Chen, Jiann-Jong Shen, Ming-Ling Kuo

**Affiliations:** ^1^Department of Microbiology and Immunology, Graduate Institute of Biomedical Sciences, College of Medicine, Chang Gung University, Taoyuan, Taiwan; ^2^School of Traditional Chinese Medicine, College of Medicine, Chang Gung University, Taoyuan, Taiwan; ^3^Division of Allergy, Asthma, and Rheumatology, Department of Pediatrics, Chang Gung Memorial Hospital, Taoyuan, Taiwan; ^4^Department of Pediatrics, New Taipei Municipal TuCheng Hospital, New Taipei City, Taiwan; ^5^Research Center for Chinese Herbal Medicine, College of Human Ecology, Chang Gung University of Science and Technology, Taoyuan, Taiwan

## Abstract

Asthma is a T helper 2 (Th2) cell-associated chronic inflammatory diseases characterized with airway obstruction, increased mucus production, and eosinophil infiltration. Conventional medications for asthma treatment cannot fully control the symptoms, and potential side effects are also the concerns. Thus, complement or alternative medicine (CAM) became a new option for asthma management. Ding Chuan Tang (DCT) is a traditional Chinese herbal decoction applied mainly for patients with coughing, wheezing, chest tightness, and asthma. Previously, DCT has been proved to improve children airway hyperresponsiveness (AHR) in a randomized and double-blind clinical trial. However, the mechanisms of how DCT alleviates AHR remain unclear. Since asthmatic features such as eosinophil infiltration, IgE production, and mucus accumulation are relative with Th2 responses, we hypothesized that DCT may attenuate asthma symptoms through regulating Th2 cells. Ovalbumin (OVA) was used as a stimulant to sensitize BALB/c mice to establish an asthmatic model. AHR was detected one day before sacrifice. BALF and serum were collected for immune cell counting and antibody analysis. Splenocytes were cultured with OVA in order to determine Th2 cytokine production. Lung tissues were collected for histological and gene expression analyses. Our data reveal that DCT can attenuate AHR and eosinophil accumulation in the 30-day sensitization asthmatic model. Histological results demonstrated that DCT can reduce cell infiltration and mucus production in peribronchial and perivascular site. In OVA-stimulated splenocyte cultures, a significant reduction of IL-5 and IL-13 in DCT-treated mice suggests that DCT may alleviate Th2 responses. In conclusion, the current study demonstrates that DCT has the potential to suppress allergic responses through the reduction of mucus production, eosinophil infiltration, and Th2 activity in asthma.

## 1. Introduction

Asthma is a chronic inflammatory disease that has several typical symptoms, such as bronchial hyperreactivity, mucus overproduction, airflow obstruction, and immune cells infiltration into the peribronchial and perivascular, especially eosinophils or neutrophils [1]. Asthma has been considered as a T helper 2 (Th2) cell-associated inflammatory diseases. Th2-related cytokines, such as interleukin- (IL-) 4, IL-5, and IL-13, are important for the development of asthma [2]. IL-4 is responsible for B cell class switching for IgE production [3]. Antigen-specific IgE further binds to IgE receptor (Fc*ε*RI) on mast cells and activates mast cells to release histamine or leukotriene [4, 5]. On the other hand, IL-5 is indispensable for eosinophil activation, maturation, and survival [6]. The mature eosinophils are navigated into the lung by chemokines, causing eosinophilia [7]. IL-13 is involved in many features of asthma. First, it enhances goblet cell differentiation, elevates bronchial hyperresponsiveness, and assists B cells class switch to IgE [8]. IL-13 can also trigger bronchial smooth muscle contraction by activating STAT6 signaling and increasing RhoA expression [9]. Nevertheless, IL-13 also enhances *muc5ac* expression and triggers mucus hypersecretion [10]. Thus, these Th2-related cytokines contribute important roles in the development of asthma and are considered as drug targets for asthma treatment.

For asthmatic treatments, *β*2-agonists and corticosteroids are common treatments for asthma. According to the Global Initiative for Asthma (GINA) report, it is now recommended that inhaled corticosteroids (ICS) containing treatment should be administrated for adult and adolescents with asthma [11]. However, adverse effects (AE) such as adrenal crisis, osteoporosis, and respiratory infections have been mentioned in the use of ICS [12]. Abuse usage of corticosteroids may even increase mortality rate [13]. Thus, personalized medicine with more specific therapeutic approaches was developed to reduce AEs from corticosteroids, also for steroid-resistant asthmatic patients. Antibodies for neutralizing IL-4, IL-5, IL-13, and IgE were considered as personalized medicine to improve asthma patient status [14]. Despite the usage of antibodies are commonly used in severe asthmatic patients, different herbal medicines can be also applied to different stages due to their therapeutic mechanisms [15]. For example, *Glycyrrhiza uralensis* was demonstrated to have steroidal effects to reduce Th2 responses and LPS-induced inflammation, which can be applied to step 2 patients in the GINA report [16]. *Platycodon grandifloras and Bupleurum chinense* suppress IL-17-related responses, which are related to neutrophilic asthma and may be administrated to support patients in step 5 [17, 18]. Herbal medicines can also be conducted into decoctions for therapeutic approach. For asthma, Xiao-Qing-Long-Tang [19] and Ma Huang Tang [20] have been reported to reduce airway hyperresponsiveness (AHR), IgE production, or eosinophil infiltration in allergen-induced asthmatic models. Also, Pingchuan formula has been reported to restore Treg/Th17 balance and downregulate the TLR2-ERK signaling pathway to reduce asthmatic symptoms [21, 22]. Thus, herbal decoctions become one of the options for asthma treatment.

Ding Chuan Tang (DCT) is a commonly used herbal decoction and routinely prescribed to patients having coughing, wheezing, and chest tightness [23]. DCT is composed with *Ginkgo biloba*, *Ephedra sinica*, *Tussilago farfara*, *Morus alba*, *Pinellia ternata*, *Perilla frutescens*, *Prunus armeniaca*, *Scutellaria baricalensis*, and asthmatic *Glycyrrhiza uralensis*. Previously, our study demonstrated that DCT improved children's AHR in a randomized and double-blind clinical trial [24]. However, the mechanism of how DCT relieves asthma remains unclear. In the present study, we investigated whether DCT can attenuate asthma symptoms through mediating Th2 activity, eosinophil infiltration, or mucus production in ovalbumin- (OVA-) sensitized mice model.

## 2. Methods

### 2.1. Ding Chuan Tang Preparation

Ding Chuan Tang (DCT) decoction was prepared and gifted by Sun Ten Pharmaceutical Company (Taipei, Taiwan), batch no. RT13425112501, and prepared as previously described [24]. Briefly, a mixture of 997 g of the nine components, including *Ginkgo biloba* 200 g, *Ephedra sinica* 133 g, *Tussilago farfara* 133 g, *Morus alba* 133 g, *Pinellia ternata* 133 g, *Perilla frutescens* 83 g, *Prunus armeniaca* 66 g, *Scutellaria baricalensis* 66 g, and *Glycyrrhiza uralensis* 50 g, was soaked and boiled in 12 L of water. After boiling for 60 minutes, the decoction was filtered and concentrated into 600 ml for further experimental usage.

### 2.2. Reagents

Ovalbumin (OVA) and methacholine (Mch) (Sigma-Aldrich, Saint Louis, MO, USA) and aluminum hydroxide gel (Pierce Biotechnology Inc., Rockford, IL, USA) were applied for OVA-sensitization experiments. RPMI 1640, fetal bovine serum (FBS), and penicillin/streptomycin (Gibco, Life Technologies, Grand Island, NY, USA) were used for splenocyte cultures.

### 2.3. Experimental Animals

Female BALB/c mice were purchased from National Laboratory Animal Center (Taipei, Taiwan) and were maintained in an animal facility under standard laboratory conditions for at least 1 week prior to the experimental use. All animal experiments were performed according to the guidelines of Animal Care Committee of Chang Gung University and NIH Guides for the Care and Use of Laboratory Animals (IACUC approval number CGU14-194).

### 2.4. OVA Sensitization/Challenge and DCT Treatment

**Figure 1:**
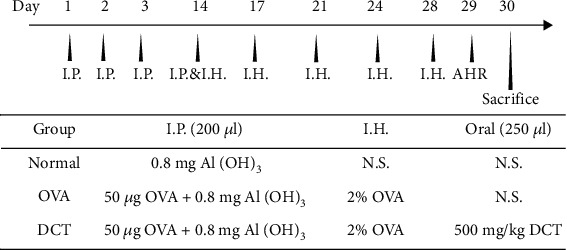
Experimental procedure for OVA sensitization and Ding Chuan Tang treatment. Eight-week-old mice were sensitized by intraperitoneal (I.P.) injection of 50 *μ*g ovalbumin (OVA) mixed with 0.8 mg of aluminum hydroxide (Al(OH)_3_) gel in a total volume of 200 *μ*l on days 1, 2, 3, and 14. The mice were then challenged by inhalation (I.H.) of 2% aerosolized OVA on days 14, 17, 21, 24, and 28. OVA was replaced by normal saline (N.S.) in the normal control group. DCT (500 mg/kg) in 250 *μ*l was given orally from day 14 to 28. N.S. was given to the normal control group and OVA-challenged group for comparison. Airway hyperresponsiveness (AHR) was determined on day 29, and mice were sacrificed 24 hours later. I.P.: intraperitoneal; I.H.: inhalation; N.S.: normal saline; DCT: Ding Chuan Tang.

All mice were randomly divided into three groups: normal control (N), OVA-challenged (OVA), and DCT-treated OVA-challenged (DCT) groups. The sensitization and treatment protocols are summarized in Figure 1. Briefly, 8-week old mice were sensitized by intraperitoneal (I.P.) injection of 50 *μ*g OVA mixed with 0.8 mg of aluminum hydroxide gel in a total volume of 200 *μ*l on days 1, 2, 3, and 14. These mice were then challenged with aerosolized 2% OVA in normal saline (N.S.) using an ultrasonic nebulizer (DeVilbiss Pulmo-Aide 5650D, USA) on days 14, 17, 21, 24, and 28, while the normal control group received N.S. only. For DCT administration, 500 mg/kg of DCT was diluted in 250 *μ*l N.S. and orally administrated from day 14 to day 28. N.S. was provided to the normal control group and OVA-challenged group for comparison. AHR was analyzed on day 29, and mice were sacrificed 24 hours later.

### 2.5. Airway Hyperresponsiveness (AHR) Response

AHR of each mouse was determined on day 29 triggered by methacholine (Mch). Mice were exposed to aerosolized N.S. or increasing concentrations of Mch (10, 20, 30, and 40 mg/ml) for 3 minutes. The enhanced pause (Penh) value was measured for 3 minutes, and pulmonary airway obstruction was analyzed by BioSystem XA software (Buxco Electronics, Inc., Wilmington, NC, USA). Penh value was calculated as follows: Penh = (TE/RT − 1)∗(PEF/PIF), TE stands for expiratory time, RT stands for relaxation time, PEF stands for peak expiratory flow, and PIF stands for peak inspiratory flow.

### 2.6. Bronchoalveolar Lavage Fluid (BALF) Collection and Cell Composition

Mice were sacrificed on day 30, and serum samples were collected for antibody detection. The trachea of each mouse was cannulated, and the lung was lavaged 3 times of 1 ml normal saline. Total cell counts were determined by trypan blue dye (Biological Industries, Beit-Haemek, Israel) exclusion. The first 1 ml lavage fluid was collected and centrifuged with 1500 rpm, 5 minutes. Supernatant was collected and stored at -80°C for further analyses. Cell pellets were then resuspended into the rest of the lavage fluid. For differential cell counts, BALF cells were centrifuged (Shandon Cytospin 4; Thermo Scientific, Pittsburg, PA, USA) onto the slides and stained with Liu staining solution (Tonyar Biotech Inc., Taoyuan, Taiwan). Different cell types were distinguished under light microscopy by counting at least 200 cells based on their morphological profiles.

### 2.7. Histological Assessment of Lung Tissue

Lung tissues were harvested and fixed with 4% formaldehyde for 24 hours at 4°C for histological examination. After dehydration, the tissues were embedded in paraffin and sliced into 4 *μ*m sections. The tissue sections were stained by hematoxylin and eosin (H&E), periodic acid-Schiff (PAS) stains, or eosinophil-mast cell stain kit (Abcam Inc. Cambridge, MA, USA). The quantitative analyses of inflammatory cell infiltration and goblet cell hyperplasia were determined by automated MetaMorph microscopy and MetaMorph software, which can distinguish different types of inflammatory cells by changing the threshold of the colors if the cells were stained with different colors.

### 2.8. Splenocyte Isolation and OVA Stimulation

Spleens were obtained from mice at the end of experiments. Single cell suspension was harvested by gently grinding spleens using frosted slides. Cells (5 × 10^6^ cells/ml) were cultured in RPMI 1640 medium containing 10% FBS and 1% penicillin/streptomycin without or with 100 *μ*g/ml OVA at 37°C, 5% CO_2_. Culture supernatants were harvested on day 6.

### 2.9. Analysis of Cytokine Concentration and Serum OVA-Specific IgE Levels

IL-4, IL-13, and IFN-*γ* levels in splenocyte cultures were determined by ELISA Duoset kit (R&D System Inc., Minneapolis, MN, USA), and IL-5 levels were measured by mouse IL-5 ELISA kit (BD PharMingen, San Diego, CA, USA), according to the manufacturer's instructions. For the levels of OVA-specific IgE antibodies, 10 *μ*g/ml of OVA was coated onto the plates (Costar, Corning, NY, USA), blocked by 1% bovine serum albumin (BSA) (Sigma-Aldrich), and then incubated with serum samples. Next, biotinylated rat anti-mouse IgE monoclonal antibodies (BD PharMingen), streptavidin-conjugated horseradish peroxidase (HRP) (BD PharMingen), and TMB substrate solution (BD PharMingen) were sequentially added to the plates. The reaction was stopped with 2 N H_2_SO_4_. Absorbance was measured on an ELISA reader at 450 nm (SpectraMax M2 Molecular Devices, CA, USA).

### 2.10. RNA Isolation and Quantitative Real-Time PCR Analysis

Total RNA was extracted from homogenized lungs using TRIzol reagent (Invitrogen, Life Technologies, Carlsbad, CA, USA). The cDNA was generated using random primers (Thermo Fisher Scientific Inc., Waltham, MA, USA) and M-MLV reverse transcriptase (Invitrogen, Life Technologies). Real-time PCR was performed using SYBR Green Master Mix (Thermo) and amplified by the CFX Connect Real-Time PCR system (iQ5, Bio-Rad Laboratories, Inc., Hercules, CA, USA). The specific primers of mouse sequences for *β*-actin, muc5ac, and gob5 qPCR were listed on Supplementary Table 1. Real-time PCR reaction conditions were 95°C for 10 minutes, then 40 cycles of 95°C for 15 seconds, and 60°C for 1 minute. The relative expression of each gene was calculated by normalizing the level to the expression of *β*-actin and demonstrated as fold change, as compared with the normal control group.

### 2.11. Statistical Analysis

Results are presented as mean ± SEM. Significance was calculated by unpaired *t* test using Mann–Whitney test. *p* < 0.05 was considered significant. All graphs and statistical analysis were generated using GraphPad Prism 8.1 software.

## 3. Results

### 3.1. DCT Alleviates Airway Hyperresponsiveness in OVA-Sensitized Mice

**Figure 2:**
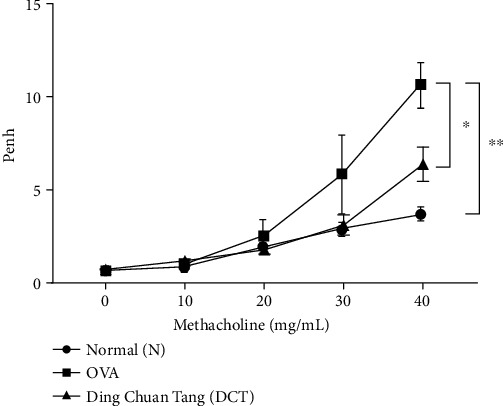
DCT attenuates airway hyperresponsiveness (AHR) in OVA-challenged mice. AHR was assessed by inhaling sequentially increased doses of methacholine (Mch) on day 29. Data are represented as Penh values and shown as mean ± SEM (*n* = 8 for normal group, *n* = 10 for OVA group and DCT group, ^∗^*p* < 0.05, ^∗∗^*p* < 0.01 compared to OVA-challenged group).

To investigate whether Ding Chuan Tang (DCT) can alleviate asthma symptoms, we established an OVA-sensitization asthmatic model and treated mice with DCT (Figure 1). Mice were sensitized with OVA for three days and then challenged with OVA by inhalation five times within the last two weeks. For treatment, mice received DCT daily from day 14 to 28. Airway hyperresponsiveness (AHR) of each mouse was detected one day before sacrifice by mice inhaling sequentially increased methacholine (Mch) for characterizing the severity of asthma. When Mch was applied, Penh values were increased in OVA-induced allergic mice, compared to the normal group. The treatment of DCT significantly reduced Penh value when mice inhaled 40 mg/ml Mch (*p* < 0.05) (Figure 2), indicating that DCT treatment could attenuate AHR in OVA-allergic mice.

### 3.2. DCT Significantly Reduces Eosinophil Infiltration in OVA-Challenged Mice

**Figure 3:**
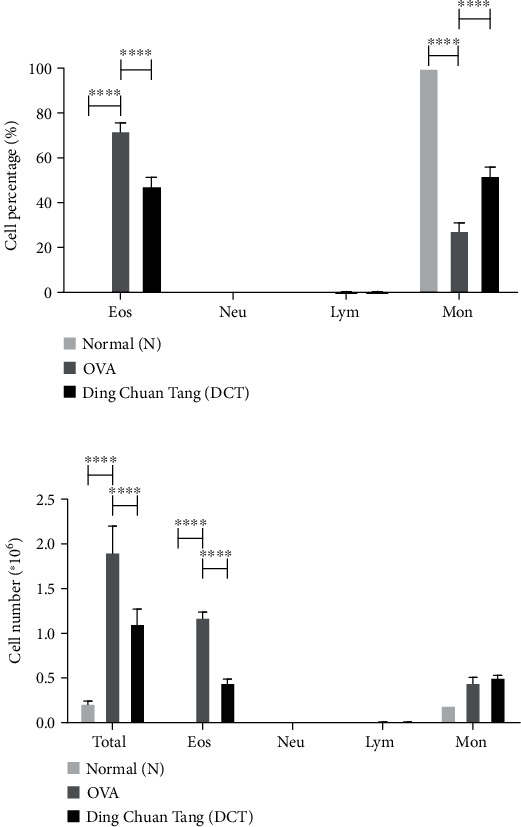
DCT significantly decreases eosinophils recruitment and total cell infiltration in BALF. The (a) percentage and (b) total or differential cell counts in BALF were determined by Liu's staining. Eos: eosinophils; Neu: neutrophils; Lym: lymphocytes; Mon: monocytes. Data are shown as mean ± SEM (*n* = 8 for normal group, *n* = 10 for OVA group, *n* = 10 for DCT group, ^∗∗^*p* < 0.01, ^∗∗∗^*p* < 0.001 compared to OVA-challenged group).

**Figure 4:**
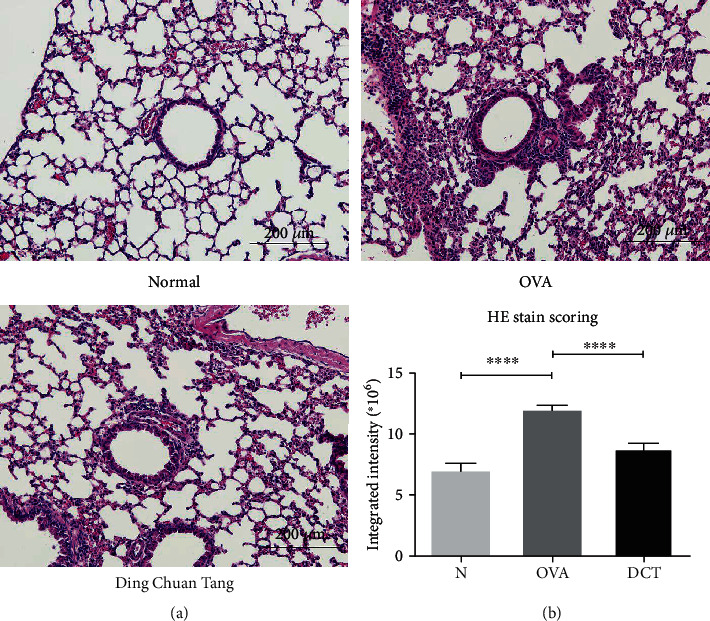
DCT alleviates inflammatory cell infiltration in peribronchial and perivascular site. Lung tissues from different groups of mice were fixed with formaldehyde. Paraffin-embedded lung tissues were sliced and stained with hematoxylin and eosin (H&E). (a) One representative section is shown for each mouse group (scale bar = 200 *μ*m). (b) The quantitative results of the H&E staining were analyzed by MetaMorph and expressed as integrated intensity. Data are shown as mean ± SEM (20 sections for each group, ^∗∗∗∗^*p* < 0.0001 compared to OVA-challenged group).

The accumulation of eosinophils in the allergic lungs is another important feature for asthma. We collected bronchoalveolar lavage fluid (BALF) from each mouse after sacrifice and analyzed leukocyte distribution in BALF. The percentage of eosinophils was severely increased after OVA sensitization, but was significantly reduced after DCT treatment (Figure 3(a)). Neutrophils and lymphocytes were nearly not detected, while the percentage of monocytes was significantly recovered after DCT treatment. The total cell counts in BALF also demonstrated DCT can significantly reduce cell infiltration in the lungs of asthmatic mice (Figure 3(b)). In the DCT group, the reduction of infiltrated cells was mainly contributed by the reduced eosinophils since other leukocytes were maintained at comparable levels. Furthermore, the results of H&E staining indicated a remarkable cell infiltration in peribronchial and perivascular sites in the lungs of OVA-challenged mice. The infiltration of cells was significantly reduced, to the similar degree as the normal control mice, after DCT treatment (Figure 4). Eosinophil-mast cell stain kit results demonstrated similar results (data not shown). These results suggest that DCT can moderate cell infiltration in asthmatic model mice.

### 3.3. DCT Significantly Reduces Mucus Production in OVA-Challenged Mice

**Figure 5:**
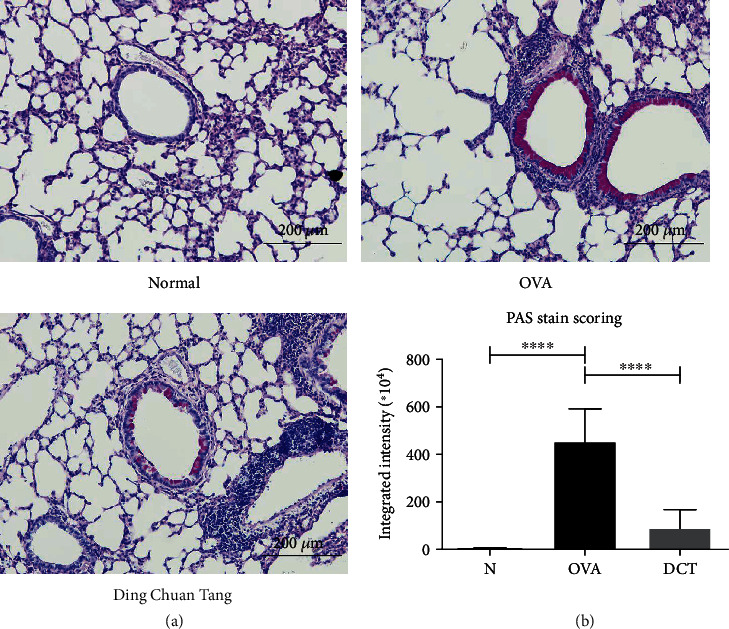
DCT reduces mucus accumulation in peribronchial site. Lung tissues were harvested and formaldehyde fixed from different groups of mice. Paraffin-embedded lung tissues were sliced and stained with PAS stain. (a) One representative section is shown for each group. Scale bar = 200 *μ*m. (b) The quantitative results of the PAS staining. Data are shown as mean ± SEM (20 sections for each group, ^∗∗∗∗^*p* < 0.0001 compared to OVA-challenged group).

Mucus overproduction is another important feature in asthma. Therefore, we performed PAS staining onto pulmonary histopathology slides to illustrate whether DCT can reduce mucus production. The results demonstrated that mucus production was conspicuous in OVA-challenged mice, while DCT significantly attenuated the accumulation level of mucus in peribronchial areas (Figure 5). Mucus was not detectable in nonsensitized mice. We further examined the expression of *gob5* and *muc5ac* genes. DCT treatment reduced the expression of *gob5* gene with approximated significance (*p* = 0.0571), but did not significantly affect the expression of *muc5ac* gene (Supplementary Figure 1). Thus, DCT treatment might relieve asthmatic symptoms with the reduction of mucus production and goblet cell hyperplasia in OVA-sensitized mice.

### 3.4. DCT Treatment Downregulated IL-5 and IL-13 Production from OVA-Stimulated Splenocytes

**Figure 6:**
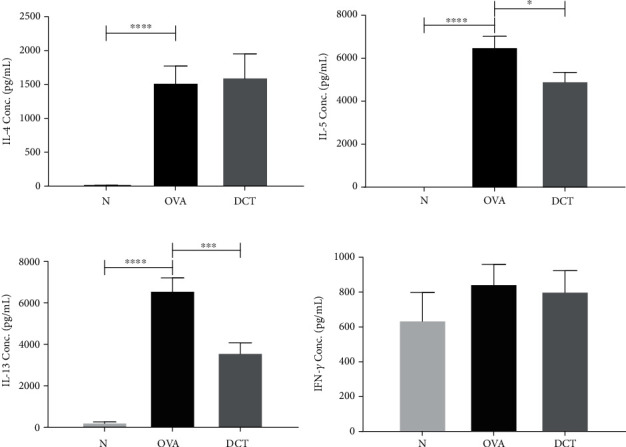
DCT reduces IL-5 and IL-13 in OVA-stimulated splenocyte cultures. (a) IL-4, (b) IL-5, (c) IL-13, and (d) IFN-*γ* cytokine levels in the supernatants of OVA-stimulated splenocyte cultures were determined by ELISA. Data are shown as mean ± SEM (*n* = 10 for normal group, *n* = 16 for OVA group, *n* = 15 for DCT group, ^∗^*p* < 0.05, ^∗∗^*p* < 0.01, ^∗∗∗∗^*p* < 0.0001).

**Figure 7:**
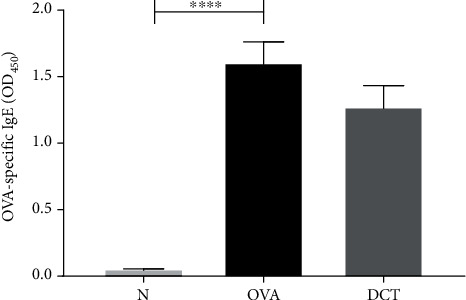
OVA-specific IgE levels in the serum of OVA mice treated without or with DCT. OVA-specific IgE level in mice serum was analyzed by ELISA. Data are shown as mean ± SEM (*n* = 10 for normal group, *n* = 16 for OVA group, and *n* = 15 for DCT group).

To dissect the influence of DCT on Th2 responses, we collected splenocytes from mice and stimulated with OVA for 6 days. Supernatants were collected for cytokine detection. The concentrations of Th2-related cytokines, IL-4, IL-5, and IL-13 were significantly elevated in OVA-induced asthmatic mice. DCT treatment significantly suppressed the secretion of IL-5 and IL-13 in splenocyte cultures, but did not affect the levels of IL-4 and IFN-*γ* (Figure 6). In addition, slightly decreased OVA-specific IgE in serum was detected after DCT treatment (Figure 7). These results suggest that IL-5 and IL-13 may play important roles in how DCT alleviates asthmatic symptoms.

## 4. Discussion

Asthma is a high prevalent chronic respiratory disease which influenced at least 350 million people accompanied with high economic burden [25, 26]. Corticosteroids, leukotriene receptor antagonists, *β*_2_-agonists, anti-IgE, or anti-IL-5 are common therapies for asthma patients, while, even if inhaled corticosteroids are the recognized fundamental anti-inflammatory treatment for achieving asthma control, abuse usage of inhaled corticosteroids may increase the risk of pneumonia and cause invasive pneumococcal diseases [27, 28]. Also, the costs of the above treatments are high and are not always available in some areas. Alternatively, traditional Chinese medicine (TCM) or acupuncture has been routinely used to support the control of asthma [29–31]. For example, antiasthma herbal medicine intervention (ASHMI) is the herbal remedy that has been approved by US FDA for clinical trials [32]. ASHMI is modified from MSSM-02, a combination of 14 herbs, and can reduce eosinophil infiltration, mucus and collagen production, and Th2-related cytokines by inducing Th1 responses [33, 34]. Another commonly used remedy for asthma, Xiao Qing Long Tang, was more likely to suppress neutrophilic asthmatic responses through neurotrophin regulation [19]. In this study, we demonstrated that Ding Chuan Tang (DCT) inhibits OVA-induced airway inflammation and eosinophil infiltration with the suppression of Th2-related immune responses in asthmatic mice, especially IL-5 and IL-13 responses.

Airway inflammation, cell infiltration, and mucus production act as key determinants of the response to stimuli and cause bronchial construction and airflow obstruction [35]. In asthma, eosinophils are predominant cells in lung tissues, while they contribute to tissue damage and airway hyperresponsiveness (AHR) in asthma [36, 37]. We observed significant AHR response and eosinophil infiltration in mice after receiving OVA, whereas DCT treatment efficiently ameliorated this phenomenon. This result was consistent with the H&E histological staining whereas inflammatory scoring was significantly decreased with DCT treatment. The activity of CD4^+^ Th2 lymphocytes dominates the response in typical allergic asthma [38]. IL-5 induces maturation of eosinophils, prolongs eosinophil survival, and enhances eosinophil effector functions [39]. IL-13, on the other hand, promotes the survival and migration of eosinophils, increases mucus production by airway epithelial cells, and elevates AHR [40, 41]. The downregulation of IL-5 and IL-13 in OVA-stimulated splenocyte cultures supports the decreased eosinophil infiltration in BALF and mucus production in the lung tissues, respectively. Also, the attenuation effect of DCT on reducing eosinophil numbers was similar to inhaled corticosteroids within normal or chronic asthmatic murine models [42, 43]. IL-4 is another important Th2 cytokine, and the major function is to promote B cell class switching leading to elevated serum IgE levels [44]. However, the secretion of IL-4 in the supernatants of OVA-stimulated splenocyte cultures was not affected by DCT treatment. The discrepancy for the effect of DCT on IL-4 and IL-5 production could be due to the heterogenic cytokine production in different Th2 cells [45, 46] or the distinguish transcription factor for IL-4 or IL-5 gene expression [47, 48]. Together with the comparable IL-4 expression between control and treated mice, serum OVA-specific IgE level was only slightly reduced in DCT-treated mice. These data consist with our previous clinical study in which asthma patients receiving DCT had similar serum IgE concentration but had improved airway responsiveness upon methacholine challenge [24]. Compounds within DCT might attenuate airway hyperresponsiveness through inhibiting mast cell degranulation and granule translocation [49, 50], since mast cells are known to play central roles in asthma airway hyperresponsiveness activity [51].

Since bronchial asthma is triggered by multiple factors, the herb decoction has the potential to target different inflammatory pathways simultaneously [29]. The most abundant component of DCT is *Ginkgo biloba* that was previously shown to reduce epithelial thickness and the number and goblet cells in OVA-challenged mice [52]. In addition, the ethanol extract of *Perilla frutescens* significantly suppressed IL-5 and IL-13 secretion in OVA-stimulated splenocytes [53]. Kuwanon G is a compound isolated from the root bark of *Morus alba* and can reduce OVA-specific IgE and IL-4, 5, 13 productions in allergic mice [54]. Flavonoids of *Glycyrrhiza uralensis* can also reduce eosinophils infiltration and Th2 responses [55]. Water extract of *Pinellia ternate* attenuated OVA-induced cells infiltration in lung, allergic airway inflammation, and mucus secretion in asthmatic mice [56]. Thus, these components may synergistically contribute to the decrease of cell infiltration and mucus production and improve asthma features.

## 5. Conclusions

In summary, we demonstrated that, similar to our clinical study, DCT could alleviate AHR, eosinophil infiltrations, and mucus production in OVA-induced asthmatic mice. Our results suggested that DCT might be beneficial to asthma airway inflammation through the suppression of IL-5 and IL-13 production.

## Data Availability

All data generated or analyzed during this study are included in this published article and its supplementary information files.

## Abbreviations

DCT: Ding Chuan Tang
Th: T helper
IL: Interleukin
OVA: Ovalbumin
AHR: Airway hyperresponsiveness
BALF: Bronchoalveolar lavage fluid
Ig: Immunoglobulin
CAM: Complementary and alternative medicine
Mch: Methacholine
IFN: Interferon
OVA: Ovalbumin.

## Supplementary Materials

Supplementary Materials Supplementary Figure 1: DCT alleviates mucus-related gene expressions in the lungs of allergic mice. Supplementary Table 1: primers used for real-time PCR amplification.
