# Supplementary material for: Ding Chuan Tang Attenuates Airway Inflammation and Eosinophil Infiltration in Ovalbumin-Sensitized Asthmatic Mice
Source: Biomed Res Int. 2021 Sep 20;2021:6692772. doi: 10.1155/2021/6692772 (PMC8478538; doi:10.1155/2021/6692772)
Supplement: Supplementary Materials — Supplementary Figure 1: DCT alleviates mucus-related gene expressions in the lungs of allergic mice. Supplementary Table 1: primers used for real-time PCR amplification. [file 6692772.f1.docx]

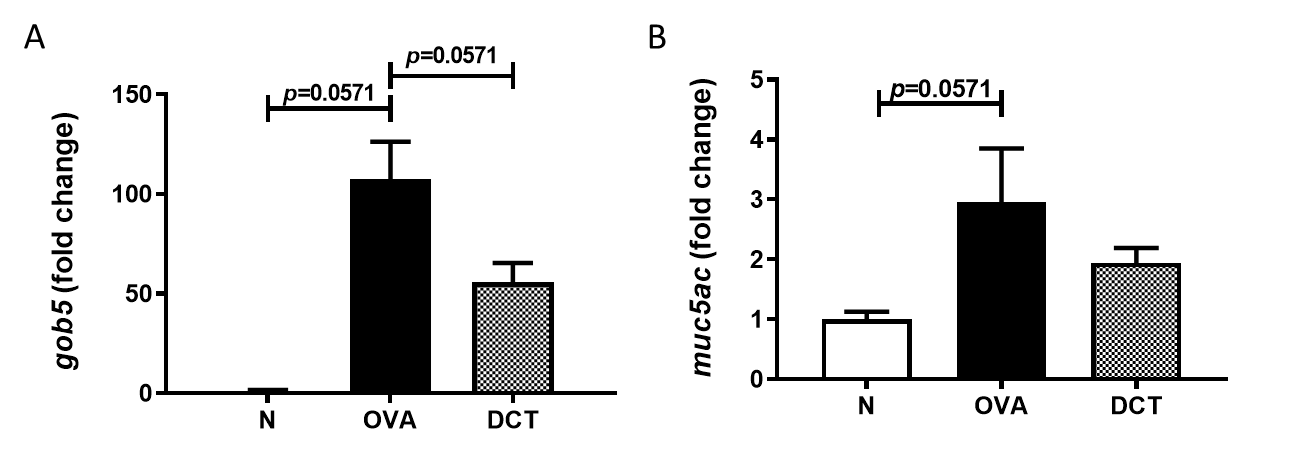


**Supplementary Fig. 1. DCT alleviates mucus-related gene expressions in the lungs of allergic mice**

The RNA expression levels of (A) *gob5* and (B) *muc5ac* genes in the lungs of mice groups. The RNA expression levels were determined by quantitative PCR and normalized to β-actin. The fold change was derived based on the comparison with normal control mice (mean ± SEM). (n=3-4 per each group, *p<0.05).

**Supplementary Table 1. Primers used for real-time PCR amplification.**

| Gene | Forward/Reverse | Sequence |
| --- | --- | --- |
| β-actin | Forward | 5’- AGAGGGAAATCGTGCGTGAC -3’, |
|  | Reverse | 5’- CAATAGTGATGACCTGGCCGT -3’ |
| Muc5ac | Forward | 5’- CATGGAGGGGACCTGGAAAC -3’ |
|  | Reverse | 5’- CCACATGGGGTCACACTTC -3’ |
| Gob5 | Forward | 5’- TCCAGGCTGTGGATAAGTCC -3’ |
|  | Reverse | 5’- TATCTTCAGCACGTGGATGC -3’ |
